# 3D Printing PLA Waste to Produce Ceramic Based Particulate Reinforced Composite Using Abundant Silica-Sand: Mechanical Properties Characterization

**DOI:** 10.3390/polym12112579

**Published:** 2020-11-03

**Authors:** Waleed Ahmed, Sidra Siraj, Ali H. Al-Marzouqi

**Affiliations:** 1 ERU and Mechanical Engineering, College of Engineering, United Arab Emirates University, Al Ain P.O. Box 15551, UAE; 2 Chemical and Petroleum Engineering Department, College of Engineering, United Arab Emirates University, Al Ain P.O. Box 15551, UAE; sidra.siraj@uaeu.ac.ae (S.S.); hassana@uaeu.ac.ae (A.H.A.-M.)

**Keywords:** PLA, silica, waste, 3D printing

## Abstract

Due to the significant properties of silica, thermostatics can be enhanced using silica-additives to maximize the quality of polymer compounds and transform plastics into tailored properties. The silica additives can enhance the handling and quality performance of composites and thermoplastic polymers due to their diverse potential. Besides, using silica as an additive in different characteristics can allow granulates and powders to flow easily, minimize caking, and control rheology. On the other hand, the eruption of 3D printing technology has led to a massive new waste source of plastics, especially the polylactic acid (PLA) that is associated with the fused deposition modeling (FDM) process. In this paper, the impact on the mechanical properties when silica is mixed with waste PLA from 3D printing was studied. The PLA/silica mixtures were prepared using different blends through twin extruders and a Universal Testing Machine was used for the mechanical characterization. The result indicated that increasing silica composition resulted in the increase of the tensile strength to 121.03 MPa at 10 wt%. Similar trends were also observed for the toughness, ductility, and the yield stress values of the PLA/silica blends at 10 wt%, which corresponds to the increased mechanical property of the composite material reinforced by the silica particles. Improvement in the mechanical properties of the developed composite material promotes the effective recycling of PLA from applications such as 3D printing and the potential of reusing it in the same application.

## 1. Introduction

**Figure 1:**
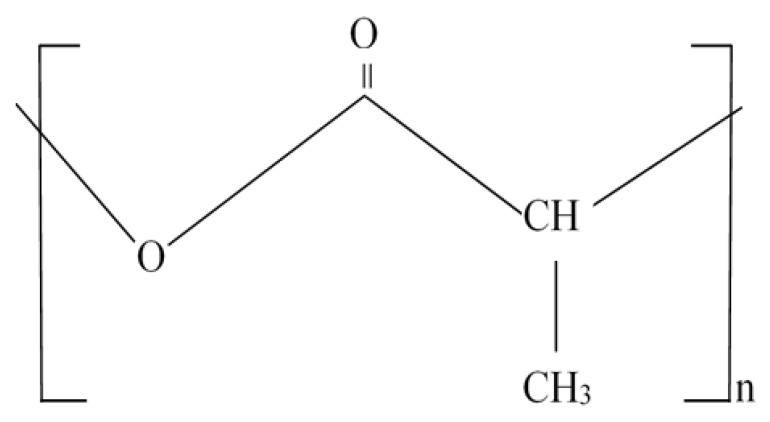
Polylactide (PLA) structure.

Consumption of plastic has increased rapidly in several fields, which has led to an increase in waste production over the past century [1]. To build a sustainable society, researchers are stimulated to work on finding environmental solutions to resolve the problem of the accumulating and the increasing waste of polymeric materials to preserve the natural resources of the earth. Out of the several bio-based polymers used, poly(lactic acid) or commonly known as polylactide (PLA) is a biodegradable polymer which is widely used in several applications such as automation, packaging, and 3D printing [2,3,4,5,6]. The ease of availability and compostability makes PLA an excellent choice for a polymeric material, however, it lacks good thermal and mechanical stability. PLA is expected to degrade at around 400 °C. However, due to its low thermal stability, it requires high efforts to recycle the material and obtain useful components from the matrices. Additionally, the degradation is accelerated due to the presence of moisture and other components such as residues of lactic acids and metal catalysts [7]. Studies on recycling PLA and improving its properties have recently increased. Figure 1 represents the structure of PLA.

Recycling polymeric waste is considered to be more practical and economical than other waste disposal techniques. The vision of the plastic economy requires innovative methods to develop sustainable solutions for manufacturing products that have high durability, prolonged shelf-life, and retain quality after recycling. Although mechanical recycling is more resource-effective, it is limited due to the presence of toxic compounds in the recycling process. However, a newer technology, which is 3D printing technology, has recently evolved as a technique that can provide sustainable production and recycling of plastics [1,8,9].

Researchers claim that since commercial PLA degrades slowly under natural conditions, it may create a negative impact on the environment due to its prolonged existence. Moreover, discarding PLA can also contribute to losing significant components such as hydroxyl groups. Therefore, it is vital to recycle PLA to reduce the consumption of renewable resources required for such monomers. Recycling PLA is highly desired as it can be used to produce eco-friendly compatibilizers, which could be used to improve the composite materials prepared from PLA, which in turn would maintain the eco-friendly nature of the material [10]. Besides, since PLA is much denser than other commodity polymers (e.g., high-density polyethylene (HDPE), low-density polyethylene (LDPE)), it can be easily separated based on its density and thus retaining the polymer would be very valuable [2].

However, due to the drawbacks of pure PLA, the addition of fillers in its matrix is preferred [11,12]. Lack of thermal and mechanical stability is one of the main drawbacks of PLA. For instance, in processes that require high processing temperatures, using pure PLA on its own would significantly affect the process as it would be unable to withstand such high temperatures even though it is a biodegradable polymer that can contribute towards sustainability. It is also reported that the addition of particulate fillers has positively influenced the mechanical strength of the composite material [7,13,14,15].

**Figure 2:**
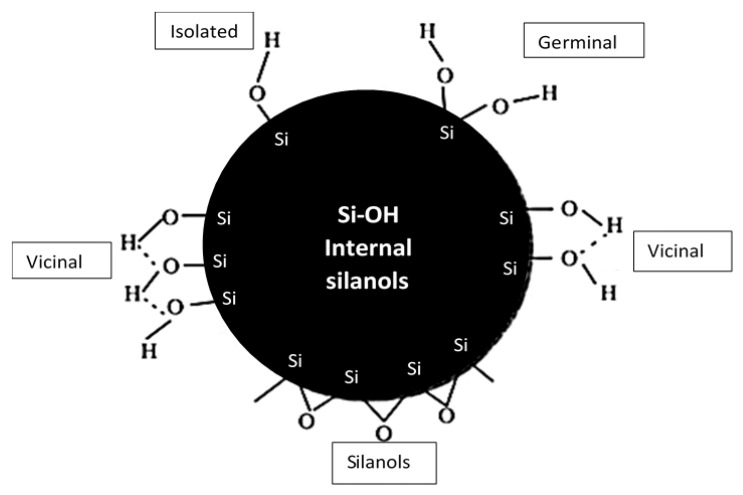
Different types of groups on the silica surface.

Filler addition is reported to enhance properties such as mechanical, thermal characteristics, and conductivity, etc., hence applications prefer the use of polymer-composite materials instead of pure polymers [16,17,18,19]. A large number of inexpensive inorganic particulate materials have been widely used as fillers to enhance the thermal and mechanical properties of polymers such as calcium carbonate (CaCO_3_), mica, kaolin clay, titania (TiO_2_), carbon nanotubes, and silica (SiO_2_) [19,20,21]. Out of the commonly used fillers, due to the high chemical and thermal stability and unreactivity, silica has been widely used in several applications to provide enhanced properties in composites. Melt blending is the most widely used technique to develop PLA/silica composites due to the process being cost-effective and simple to operate. Moreover, it is reported that the mechanical properties of the composites with filler addition could be better studied using a twin screw-extruder [19]. Literature supports that the addition of silica as reinforcement strengthens the polymer composite properties such as the elastic modulus and tensile strength [7,22,23,24,25]. Silica-based fibers are used in reinforced composite materials for increased mechanical performance [26,27,28]. Silica is incorporated with silver nanoparticles to minimize agglomerations in anti-bacterial treatments [29]. Additionally, silica is also considered a bio-safe material that can be used in the food and drug industry. It has also been reported that silica disperses well in PLA and results in improved mechanical and thermal properties, and also enhances the crystallinity of the structures [30,31,32]. Moreover, since silica is reported to have high silanol groups, siloxane bridges, and hydroxyl groups [12], it can be easily functionalized, opening pathways for several applications such as biomedical, separation, catalysis, and sensors [17,33]. Figure 2 represents different types of silanol groups and siloxane bridges on the silica surface and its internal OH groups [34].

As mentioned in the aforementioned paragraph, due to its high thermal stability and heat resistant properties, silica has also gained extensive interest in the field of 3D printing. 3D printing or fused deposition modeling (FDM) is one of the most extensive forms of fabrication due to its lowered costs, consumption of energy, and its ease of modeling and experimentation especially of intricate parts [1,9], which helps in progressive growth and expansion of knowledge in the fields, which can be of significant value, but this leads to increased 3D waste [35,36,37]. However, 3D printing can incorporate raw materials effectively in a way that can reduce waste and conserve energy. Additionally, since household waste accounts for at least 31% of the residual waste, 3D printing can include recycling systems at a household-scale level which can be considered as a good alternative to centralize the recycling [1,38].

According to a study, the biocompatibility of PLA filaments was not altered after the extrusion process, which supports the positive post-processing modifications needed for customization [39]. Moreover, silica is also known for being incorporated in scaffolds. For instance, the effect of the mechanical and biological properties of designed microporous scaffolds manufactured by commercial 3D printing technology was studied, and the results promoted the use of silica-based 3D printed scaffolds as viable candidates for further research in clinical applications [17,40]. Overall results support this technology providing an excellent technique to fabricate the small mechanical parts providing sufficient precision, which allows rapid modifications to be made during the process itself [41]. Besides, the efficiency of the process can be further improved by utilizing waste PLA as feedstock material, thereby minimizing the leftover waste PLA filaments [4,42]. Since fillers are reported to impact the mechanical properties, in this study, waste PLA from 3D printing was collected to study the impact of silica as a filler on the mechanical properties of the prepared composite material. The mechanical characteristics of the prepared PLA/silica composite material were analyzed to understand whether silica enhances the mechanical properties of the material, which could potentially be reused in the same source 3D application.

## 2. Materials and Methods

Assorted leftover PLA from 3D printing was collected from the prototyping labs of the university sorted based on the color and shredded using a shredding machine. A powerful DIY shredding machine with stainless steel blades was used in the first stage of the shredding process to break down the leftover waste material into small pieces that are used for shredding hard plastics and prepreg carbon fiber waste [43,44,45,46]. The shredded material was then broken into smaller pieces using a heavy-duty mixer that is used for hardwood, where the shredded material did not contain shredder residue [47], since the plastic waste came from lab sources and was carefully sorted.

For the sand-silica, it was collected from the local environment cleaned and dried, and was sieved to minimize the time needed for the further grinding process. The sand used in the study was collected from the identified domestic territory that is classified into three types (carbonates, silicates, and free silica, mostly in the form of detrital quartz). The analyzed mineral composition of SiO2 is reported to be approximately 36.93 wt%. Regarding the mineral content, the actual relative abundance of the minerals is as follows: silicates 47.17 wt%, carbonates 26 wt%, and quartz up to 14 wt% [48].

**Figure 3:**
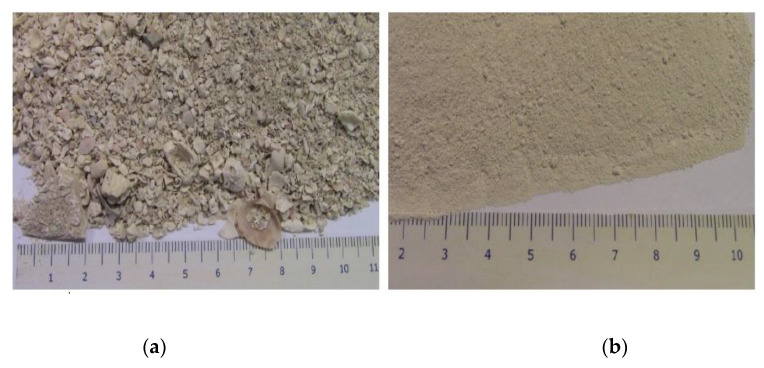
(**a**) Sample of white silicon dioxide before grinding; (**b**) Sample of the white silicon dioxide after pre-griding in PM 400.

In the next stage, the sieved particles were subjected to different stages of grinding processes under the supervision of the grinding machine manufacturer using a heavy-duty grinder (Planetary Ball Mill, PM-400, Retsch, Haan, Germany). Several grinding attempts have been carried out to try to achieve the minimum particle sizes of the silica (silicon dioxide) to mix with the PLA. The present description illustrates the successful trial that was done under dry-grinding conditions, with a pre-grinding process for 10 min and fine-grinding for 50 min. To be sure that moisture was not a reason for the caking effects, the sample was dried before grinding, as demonstrated in Figure 3.

**Figure 4:**
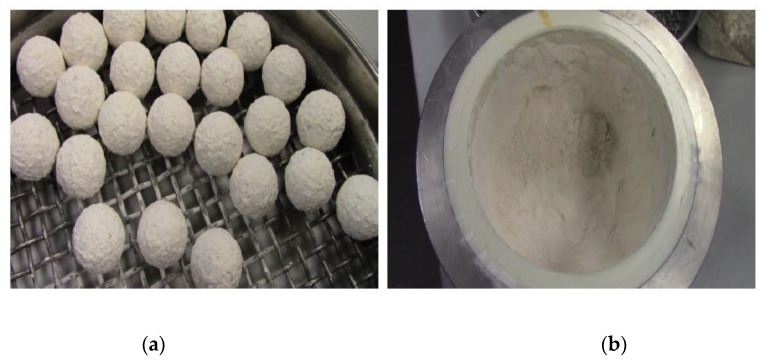
(**a**) Sample of white silicon dioxide before grinding; (**b**) Sample of the white silicon dioxide after pre-griding in PM 400.

A Planetary Ball Mill PM 400 was used to pre-grind a 250 g silica sample in the 500 mL grinding jar of Zirconia by using 8 × 30 mm grinding balls made of ZrO_2_. After 10 min at 320 rpm (interval with reverse mode) the ground silica sample was slightly caking (as shown in Figure 4), so the 30 mm balls were exchanged with 150 × 10 mm balls.

**Figure 5:**
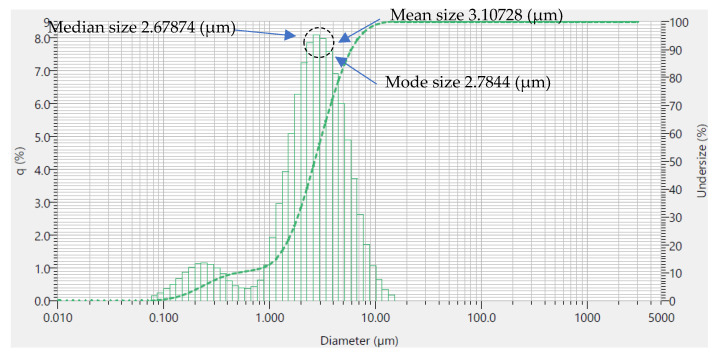
Statistical results of the Laser Scattering Particle Size Distribution Analyzer Partica LA-960 that shows the particle sizes according to ISO 13320:2009.

To reduce caking effects, the speed was reduced, and some drops of ethanol were added, followed by fine grinding with the 10 mm balls and a speed of 120 r/min, after 10 and 20 min. It was observed that the sample was only slightly caking, and after an additional 30 min, the sample was obviously caking (scratching the sample of the wall). Finally, measurements of the particle size distribution were taken in the Partica LA-960 for particle characterization (HORIBA, Ltd., Kyoto, Japan) for 10, 20, and 50 min, with grinding performed to 3 µm particle size that was verified and considered, as illustrated in Figure 5.

**Figure 6:**
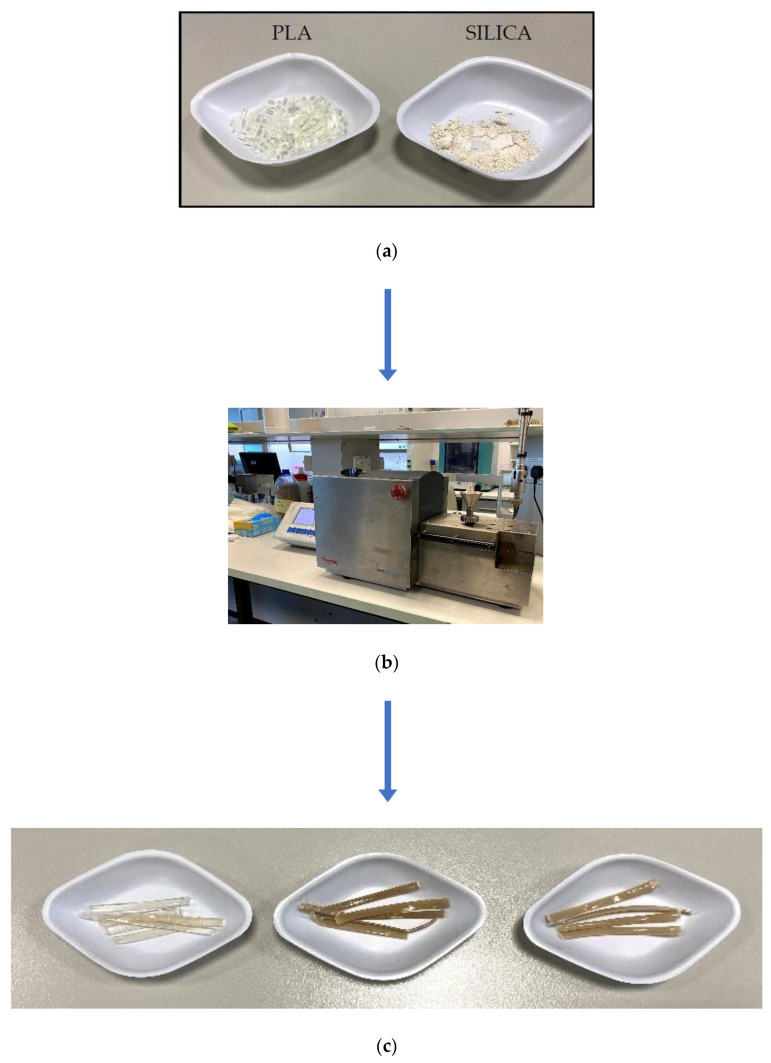
Material processing: (**a**) Shredded PLA and ground silica; (**b**) twin-screw extruder; (**c**) chopped extruded filament samples.

On the other hand, the PLA material was cut into small pieces using a mechanical shredder, enough to fit into the extruder feed hopper with an approximate length of 10 mm. In this investigation, we adopted three different mixing ratios (i.e., 5 wt%, 10 wt%, and 15 wt%) of the ground silica microparticles (~3 µm) that were later mixed with the PLA. The microparticles and the PLA were mixed at the designed ratios to produce composite samples that were developed in a melt blender using a twin-screw extruder (MiniLab HAAKE Rheomex CTW5, Karlsruhe, Germany). In general, the whole set of the mixtures, as well as the PLA alone (i.e., 0% of silica filler), were processed under a closed-loop cycle for 5 min at 190 °C with a screw rotational speed of 140 r/min to maintain the best mixing characteristics. Later, the extruder was used to blend and extrude the material out of the extruder by a valve to collect the process mixture for the next stage, as shown in Figure 6 for the material preparation process. The control sample was also prepared by blending pure PLA (0 wt%) using the same conditions as done in several other composite studies [49,50]. Due to the capabilities of the extruder that produces limited quantities of the mixtures, the extruded composites were chopped into small pieces (~1 g) and thermally compressed for 5 min using a Carver’s press (Carver™ Lab Presses) under 34.47 MPa pressure at 190 °C to produce composite sheets that were used for the mechanical investigation stage.

### Mechanical Characterization

**Figure 7:**
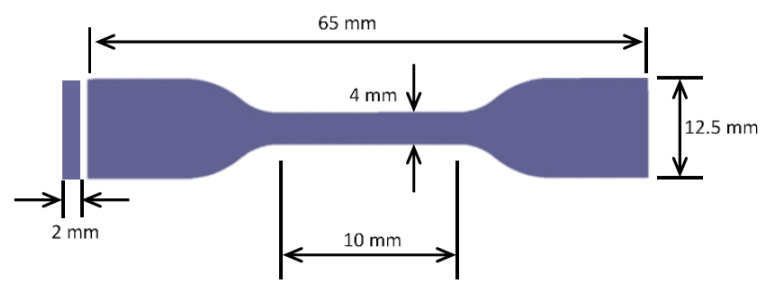
Specimen dimensions.

In order to investigate the impact of the added silica microparticles on the characteristics of the produced PLA/silica mixtures, the mechanical properties of the prepared composite sheets were tested using the Universal Testing Machine (UTM, Shimadzu, Kyoto, Japan). The results sheets from the last processing stage (i.e., thermal compression) were used to produce tensile test samples according to the American Society for Testing and Materials standard (ASTM)-D 638, as illustrated in Figure 7 with the necessary dimensions. A specialized manual blanking machine (Exacta Model-JFP, Karnataka, India) was used to prepare the tensile test sample. One of the concerns associated with this process was that the clearance between the die and the punch should be related to the thickness of the sheet to be blanked since the clearance between the punch and the die is expressed as the ratio to the thickness of the sheet [51,52]. This might affect the sheared edges and create rough surfaces that would affect the mechanical properties of the blank and possibly leading to failure. The blanking machine has fixed clearance since it is made for the thin-film blanking process, so it is expected to have a more rough surface for thicker sheets which will lead to more deterioration in the mechanical properties of the samples. Stress concentration at the edges plays a significant role in the depreciation of the mechanical properties in general. Moreover, the tool cutting edge wear would cause a similar undesirable impact that would affect eventually the roughness of the samples and hence the failure [53]. Furthermore, during the blanking process, there is significant pressure on the blanking edge of the punch and the die, which causes complex stress distribution, leading to different geometry of the intersection surface and burr size [54]

Dumbbell shape test specimens were prepared from the composite sheets of 10 (l) × 4 (w) × 0.06 (t) mm for 0 wt%, 5 wt%, and 10 wt% and of 10 (l) × 4 (w) × 0.08 (t) mm for 15 wt%. It is to be noted that the filler increases from 10 wt% to 15 wt%, which increased the thickness of the sheets. This difference in the dimension is directly associated with the processing itself. Their respective mechanical properties were studied by allying a tensile load using a tensile machine with a 10 kN load cell.

**Figure 8:**
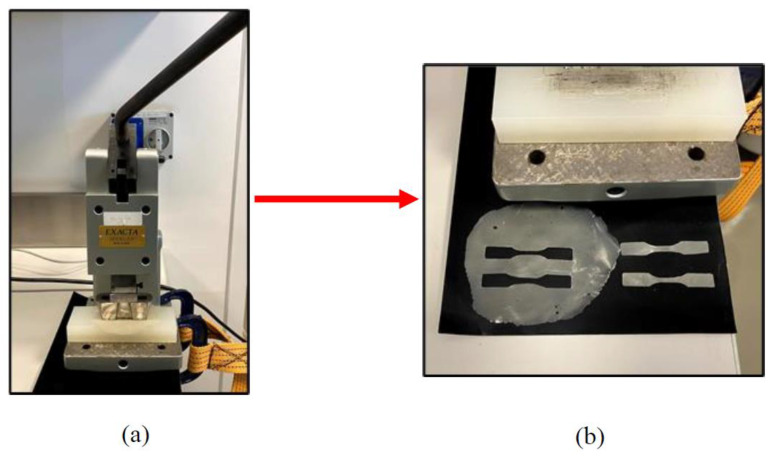
(**a**) Blanking machine; (**b**) Dumbbell shape samples after blanking process.

**Figure 9:**
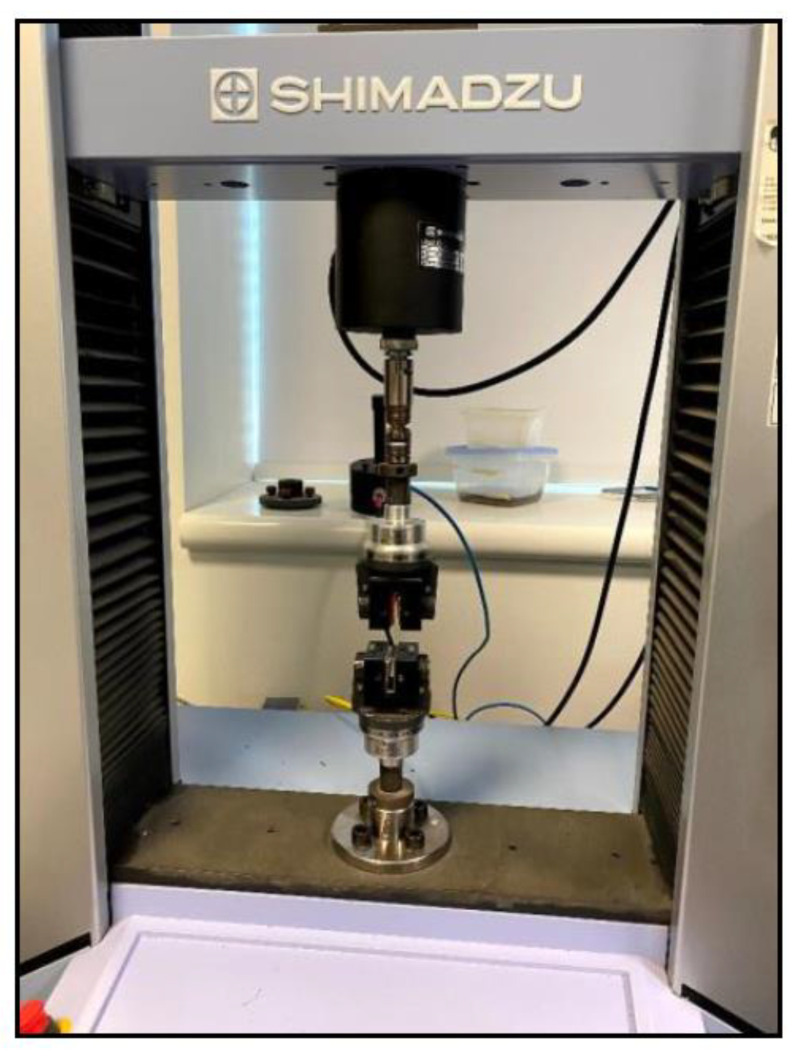
Tensile testing machine.

The rate of crosshead linear speed was set to be 5 mm/min at room temperature. A set of five repeated trials were performed for all the prepared samples. Figure 8 shows the prepared samples during the blanking process, whereas the tensile testing machine used is shown in Figure 9.

## 3. Results and Discussion

The processed material was used to produce thin sheets of the composite blends to be tested by the tensile test machine, so from each sheet, several tensile test samples were made by using a dog-bone cutting machine. The thickness of the tensile test samples was measured at three different locations along the gauge length using a precise digital thickness measurement device, and the best five samples were chosen in terms of the consistency of the thickness. Mitutoyo Thickness Gage (Model 547-526S) was used to measure the thickness of the thin film due to its high resolution (0.001 mm) and accuracy (0.0002). Average measured values were considered in this study along the gauge length of the sample [55].

**Figure 10:**
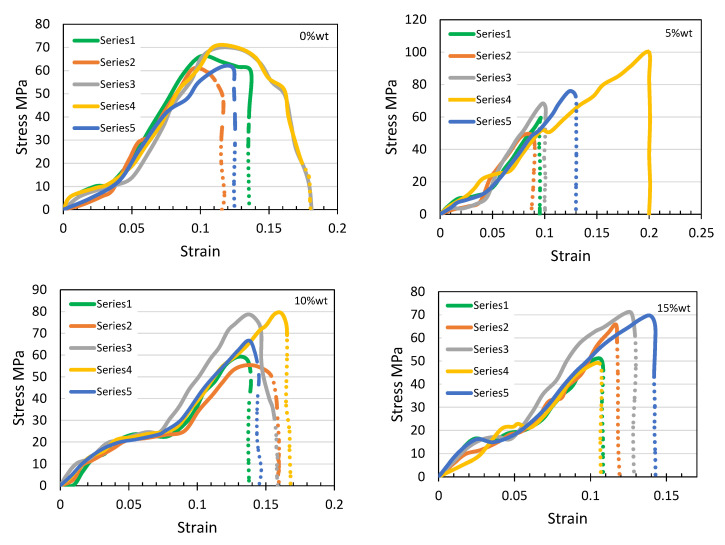
Stress-strain plot of the four composite blends.

A set of five trials were performed for each data set to obtain the mechanical properties for the data analysis. An example of the stress-strain curve is shown in Figure 10 that represents one of the samples prepared from each data set.

In order to estimate the mechanical properties of the tested samples, ASTM 638 was used. The tensile strength is calculated by dividing the measured maximum load applied to the specimen in newtons by the average original cross-sectional area of the gage length segment of the specimen in square meters. Elongation values are valid and are reported in cases where uniformity of deformation within the specimen gauge length is present. Elongation values are quantitatively relevant and appropriate for engineering design. When non-uniform deformation (such as necking) occurs within the specimen gage length nominal strain values are reported. Nominal strain values are of qualitative utility only. Percent elongation at break is estimated by reading the extension (i.e., change in gage length) at the point of specimen rupture, and then dividing the calculated extension by the original gage length and multiplying by 100. The nominal strain is the change in grip separation relative to the original grip separation expressed as a percent. Nominal strain at break is calculated at the break by reading the extension (i.e., change in grip separation) at the point of rupture, and then by dividing that extension by the original grip separation and multiplying by 100. The modulus of elasticity is calculated by extending the initial linear portion of the load extension curve and dividing the difference in stress corresponding to any segment of the section on this straight line by the corresponding difference in strain. The elastic modulus values are computed using the average original cross-sectional area in the gage length segment of the specimen in the calculations. However, for samples where no proportionality is evident, the secant value is calculated by drawing a tangent and marking off the designated strain from the yield point where the tangent line goes through zero stress. The stress used in the calculation is then determined by dividing the load extension curve by the original average cross-sectional area of the specimen.

**Figure 11:**
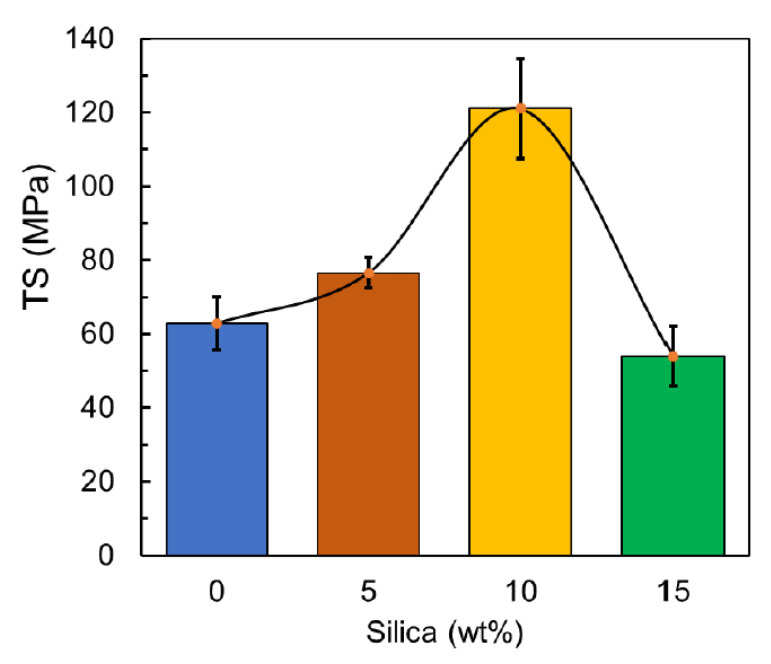
Tensile Strength values of the prepared material.

In general, it has been reported that the mechanical properties of the commercial unprocessed PLA filament are reported by the manufacturer which was used as a reference in the current experimental investigation as leftover waste [56]. Mainly, the general trend was observed that the recorded tensile strength values of the prepared PLA/silica composite increased with increasing the silica concentration, as shown in Figure 11 that reflects the positive impact of this filler on the strength resistance to the failure beyond the yield point that is attributed to the consistency to the filler spread in the matrix, where the tensile strength is estimated by Equation (1):

(1) $$Tesnile\;Sterngth\;\left(Pa\right)=\frac{Maximum\;load\;measured\;\left(N\right)}{Gauge\;length\;original\;area\;\left(m^{2}\right)}$$

Moreover, a noticeable increase in the tensile strength levels was observed from 21.9% at 5 wt% to 92.8% at 10 wt% respectively.

Beyond 10 wt%, a gradual drop in the value of tensile strength was observed which decreased to 14.1% MPa at 15 wt%. It had been observed that the TS increases when silica % increases up to 10 wt%, meaning that the impact of silica is positive to the 10 wt% mixture in terms of the material strength. After 10 wt% silica, the TS suffers from a reduction in strength that is attributed to the agglomeration of silica particles. The agglomerates act as defects and initiation spots that cause failure. Void formation in matrices is also reported to cause such behavior in filler-matrix systems [57]. Similar values and trends are reported for another PLA/silica composite study that refers to the lack of dispersion consistency of the particles in the matrix that could increase the stress concentration where the failure starts [30].

**Figure 12:**
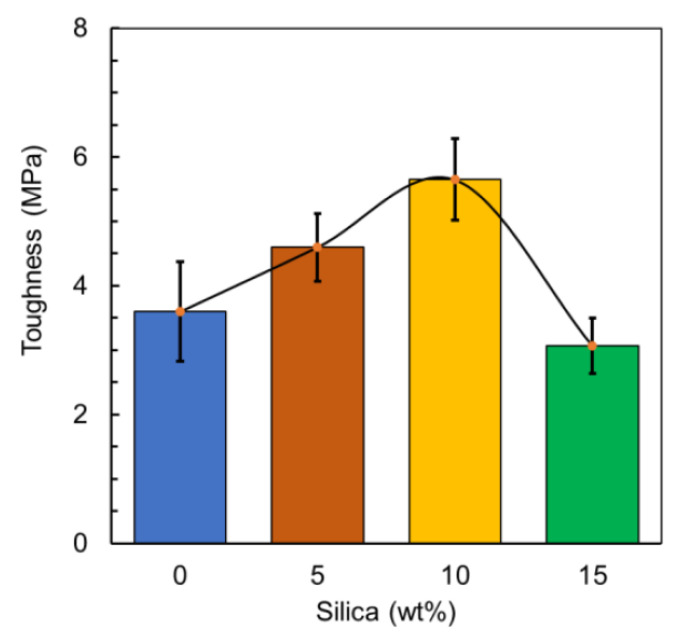
Composite toughness.

On the other hand, the toughness of the material that is illustrated in Figure 12, where toughness can be expressed by Equation (2):

Toughness = Area underneath the stress-strain (*σ*–*ε*) curve = *σ* × *ε* (2)

It has been observed that toughness increases from 3.6 MPa at 0 wt% to 5.6 MPa at 10 wt%, which means the impact of the silica increases the toughness properties of the material that is attributed to broadening the capacity to absorb energy. Effective toughening has also been reported for another silica/PLA system at 10 wt% addition [58]. A reason for this could be explained as the presence of silica particles aiding the PLA matrix to be subjected only to partial stress leading to the change in the progression of the crack direction [59,60,61]. For 15 wt% silica, the material demonstrated a significant increase in toughness to 56.9% in comparison with the reference value, which is considered the peak before a drop beyond this level, around 14.8%. This can be explained by the deboning mechanism wherein the deboning at the interphase in between the silica and PLA matrix creates voids that lead to shear yielding in the PLA matrix. Consequently, the polymer matrix can withstand higher energy and is tougher with increased filler addition. Whereas at higher concentrations, the aggregate formation can act as a flaw and reduce the toughness of the polymer composite material [34,58]. Moreover, another study conducted on a PLA/silica system demonstrated that the improved mechanical behavior depends strongly on the particle size and concentration of the silica added [62,63].

**Figure 13:**
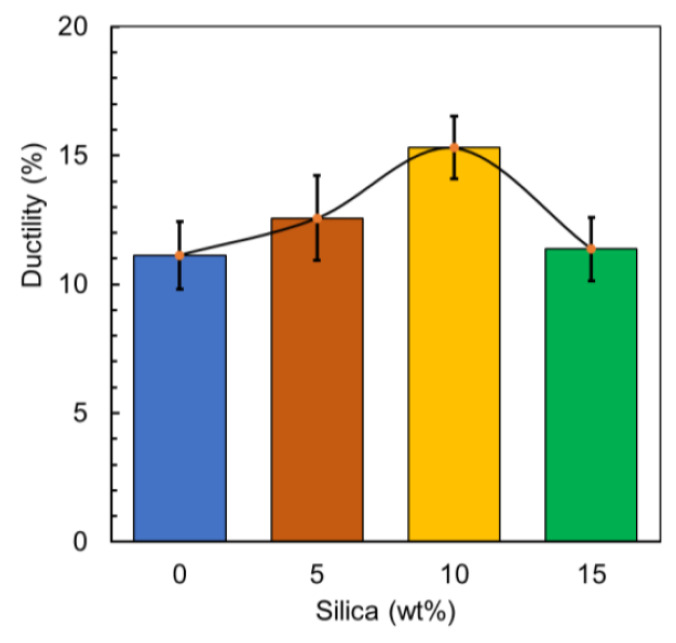
Ductility of the prepared material.

The strain at the failure point is the measure of the ductility of the material that is one of the important properties, which is the ability to deform plastically and acclimatize while being stressed with the applied load which means its attractiveness to the industrial applications that need more flexibility. It has been observed a significant increase in tested samples ductility to 12.9% for the 5% silica composite in comparison with the reference material, and demonstrated 11.1% increase in ductility when compared to the original unprocessed PLA filament [56]. Additionally, the sample prepared with 10 wt% silica addition experienced the highest increase in the ductility to 37.4%, which is demonstrated in 15.3% elongation. Beyond this, a little reduction in ductility of 2.1% was observed for the sample prepared with 15 wt%. This can be caused by the agglomeration of silica particles, resulting in stress concentrations that weaken the polymer matrix leading to failure [3]. Figure 13 represents the ductility of the prepared and tested samples that represent the statin at the failure point to stand on the developed characteristics that might be helpful for the industrial applications especially using recycled ceramic composite for 3D printing applications, that can be expressed by Equation (3):

(3) $$Ductility=\frac{L_{f}-L_{o}}{L_{o}}\times 100$$

where

*L_f_* = is the length of the specimen when it finally ruptures or breaks.
*L_o_* = is the original gauge length of the specimen

**Figure 14:**
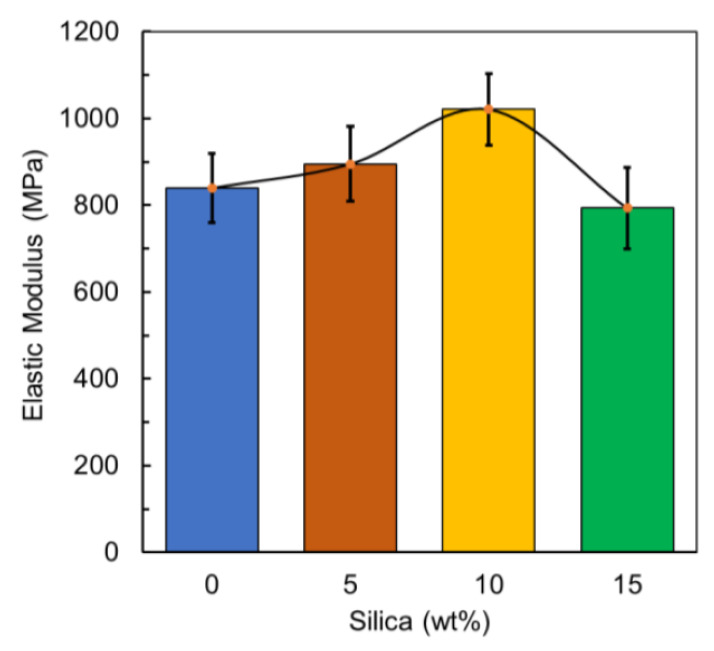
Elastic modulus blends.

The other substantial mechanical properties such as the elastic modulus and the yield stress of the prepared composite material were also analyzed. An increasing trend in both the elastic modulus and the yield stress was observed. However, for the elastic modulus, it was observed that the samples prepared with 10 wt% silica has the highest value and for the yield stress, the samples prepared with the same silica percentage showed a 21.6% and 21% increase for the both properties respectively in comparison with the reference value. The yield stress for an unprocessed PLA filament is 49.5 MPa, so in comparison, the addition of silica increased the yield stress values significantly [56]. For both moduli of elasticity and yield strength, the trend of a reduction beyond the 10% silica composite was observed and this can again be associated with agglomerate and uneven crystallization resulting in weaker bonding [3,64,65]. In general, Figure 14 represents the elastic modulus of the prepared material, where the elastic modulus (i.e., Young’s modulus) E, can be calculated using Equation (4) by dividing the tensile stress, σ(ε), by the engineering extensional strain, ε, in the elastic portion (i.e., linear) of the physical stress–strain curve:

(4) $$E=\frac{\Delta \sigma \left(\varepsilon \right)}{\Delta \varepsilon }=\frac{F/A}{\Delta L/L_{o}}=\frac{F.L_{o}}{A.\Delta L}$$

where

*E* is Young’s modulus (modulus of elasticity)
*F* is the force exerted on an object under tension.
*A* is the actual cross-sectional area, which equals the area of the cross-section perpendicular to the applied force.
Δ*L* is the amount by which the length of the object changes
*L_o_* is the original length of the object.

**Figure 15:**
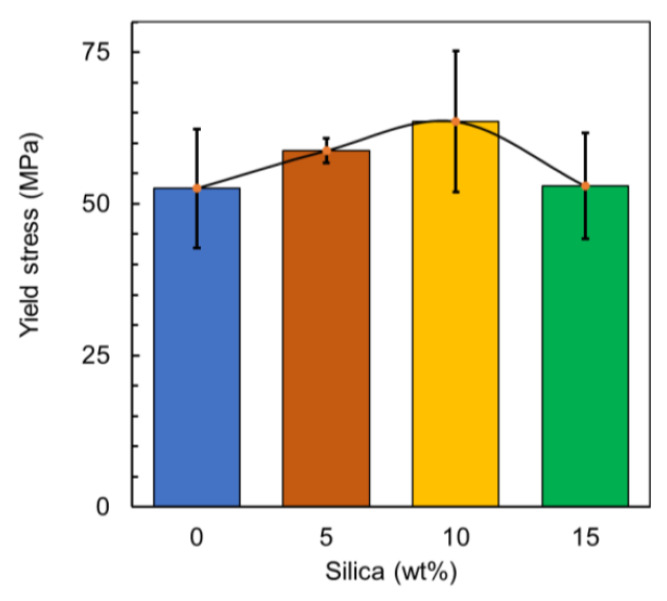
Yield stress of the prepared material.

Figure 15 demonstrates the yield stress of the prepared material, respectively. Moreover, through observation, it can be stated that a similar trend is experienced in all the properties tested except in the elastic modulus. However, variability in the results is also observed. This can be associated with the dispersion of the particles not being homogenous as it is governed by the mixing of the particles within the extrusion process itself [66]. Additionally, the shape of the particles strongly influences the inconsistent mixing of the filler [67]. The variability then occurs when the non-homogenous mixing leads to stress concentrations occurring at different locations each time leading to failures at different points. All the mechanical properties are tabulated in Table 1.

The arithmetic mean of the collected values are calculated and the standard deviation (Equation (5)) is estimated as follows:

(5) $$S=\sqrt{\left(\sum^{\;}X^{2}-n{\bar{X}}^{2}\right)/\left(n-1\right)}$$

where: *S* = estimated standard deviation, *X* = value of single reading, *n* = number of measurements, and $\bar{X}$ = arithmetic means of the set of observations.

As reported in numerous studies, an increasing trend in the mechanical properties such as in the elastic modulus and tensile strength is observed with increasing filler concentration up to 20 wt% filler addition [67,68]. As in this case, maximum mechanical properties were obtained at 10 wt% silica addition, the use of 15 wt% filler was to confirm whether the trend increases or drops.

## 4. Bulk Density

The dry density of the ground silica was estimated through the ASTM D7263–09 standard [69]. The dry density of the ground sand (i.e., *ρ_silica_*) was attained through a dried sand sample that was accomplished using a controlled drying process carried out using an electric drying oven. Using Equation (6), the sand dry density was calculated [70].

(6) $$\rho_{silica}=\frac{mass_{sand}}{Volume}$$

where *mass_sand_* is the measured mass of the dry sand and the volume is the controlled volume of the measuring cylinder used.

The apparent density of the *PLA* filament was determined according to the ASTM D792-20 standard [71]. After measuring the corresponding masses in air and water, the apparent density *(ρ_PLA_*) can be determined by Equation (7) [71].

(7) $$\rho_{PLA}=\frac{m_{air}\;\rho_{water}}{m_{air}+\;m_{water}}$$

where *ρ_water_* is the density of water, *m_air_* is the mass of the sample measured in air and *m_water_* is the mass of the sample measured in water.

In order to evaluate the composite film measured density, the theoretical density of the composite was calculated using the linear mixing rule [67], which is an approach to estimate the composite properties based on an assumption that a composite property is the volume-weighted average of the phases (matrix and dispersed phase) properties, as presented in Equation (8).

(8) $$\rho_{c}=\frac{\rho_{filler}\;\rho_{matrix}}{\rho_{matrix}\;m_{filler\;}+\;\rho_{filler}\;(1-m_{filler\;})}$$

where *ρ_c_* is the density of the composite, *ρ_filler_* is the density of the silica, *ρ_matrix_* is the density of the matrix (i.e., *PLA*), and *m_filler_* is the mass fraction of the filler. Using Equation (8) stipulates that no air inclusions are associated with the composite and that the silica filler does not influence the density of the polymer phase by nucleation of crystal growth [67].

The experimental weights of the prepared composite material were measured using a weighing balance (Citizen-CX 220, d 0.0001 g). To determine the volume, the respective test films were cut with a hand cutter according to ASTM D6287-17 standard [72], where the thickness of each composite film was measured in five spots along with the sample in order to assure thickness uniformity using Mitutoyo Thickness Gage (Model 547-526S). The collected data was then used to approximate the experimental bulk density by finding the ratio of the mass to the measured volume of the prepared samples.

**Figure 16:**
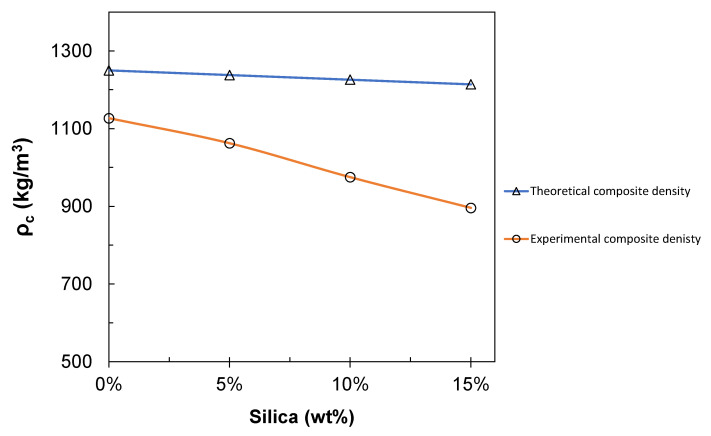
Composite density: Theoretical vs. Experimental.

Figure 16 illustrates the comparison between the theoretical density and the experimental density of the PLA/silica composite films.

The bulk density of recycled PLA filament was calculated to be 1127 kg/m^3^, which is close to the commercial value reported [73] (1250 kg/m^3^ [56]). It has been observed that the density of the composites decreased with increasing the silica content. This decrease in the value is associated with the low density of the ground silica filler used (1047 kg/m^3^). In general, there is an apparent deviation between the theoretical and experimental estimation of the composite film that starts from 9.8% for the pure polymeric case and increases up to 26.2% for the composite film with maximum silica filler. This can be attributed to the high temperatures used during the composite film processing phases (extrusion and compression). Any residual moisture or air due to poor mixing of the filler surface by the polymer is also expected to be degassed during the hot compression phase within the composite material [67]. This can further contribute to the decreasing densities due to the high viscosity of the PLA matrix [74]. Achieving decreased density can be helpful using such sorts of fillers, where low-density reinforced thermoplastic composites are useful for some commercial product applications that should have float characteristics [75]. Moreover, the material becomes easier to handle and can also be considered as one of the most important design parameters to reduce high transportation costs [76].

## 5. Conclusions

Silica is considered as one of the most abundant inorganic ceramic materials, which is composed of silicon dioxide (SiO_2_) and has unique properties that can be used as a filler to form composite materials with polymers for different applications. When silica is mixed with organic and biodegradable materials such as PLA, the resulting composite material can be used in a wide range of industrial applications, especially for the development of advanced inorganic/organic hybrid composites to achieve improved overall properties of the system and to enhance the interfacial interaction at different structural hybridization levels of organic polymers and silica fillers [19]. Furthermore, functionalized silica particles are considered as superlative fillers for a variety of polymers since silica fillers have numerous modified forms and are one of the best options to produce stable functionalities [77]. Moreover, in situ synthesized silica particles in polymeric media can give another successful way to produce hybrid silica composites for various applications [78], especially that silica particles enhance mechanical strength and overall performance of polymer/silica composites [79]. In this work, composite sheets from PLA/silica were successfully manufactured via melt blending and compression molding. Mechanical characterization of the prepared material showed an increase in the tensile strength values of the reinforced material from 0 wt% to 10 wt% as well as increases in the toughness, ductility, and yield stress concerning the original PLA waste. For instance, the peak tensile strength values and toughness increased from 62.8 MPa at 0 wt% to 121 MPa at 10 wt%, and from 3.6 MPa at 0 wt% to 5.6 MPa at 10 wt% respectively, which corresponds to the increased mechanical properties with silica reinforcement. However, beyond this point, a drop was observed. A similar trend was also observed for the ductility of the material. The increased value in ductility was around 37.4% at a silica ratio of 15 wt%, implying a similar structure in terms of the material, whereas the elastic modulus increased at 10% to 1.02 GPa. Additionally, decreasing values of elastic modulus and yield stress beyond 10 wt% for both correspond to the weakening of the composite beyond a certain point. In general, the deterioration in the mechanical properties of the particulate reinformed composite can be attributed to the interfacial characteristics between the silica particles and the matrix, as well as to the cracked particles during the production stage [80,81].

Ongoing experiments are underway on manufacturing composite sheets with increased silica concentrations to study more mechanical properties. To verify the possibility of using silica mixed with waste PLA for 3D printing purposes, further investigation would be helpful. Performing 3D printing using this reinforced PLA/silica mixture would help to understand the influence of recycling on the mechano-chemical stability of the obtained product since it would affect the 3D printing capabilities [20] of the ceramic-based material. Moreover, as a natural material, silica can be used with biomass to enhance the mechanical properties aiming to produce hybrid biodegradable composite products using 3D printing technology [82].

## Figures and Tables

**Table 1:** The mechanical properties of the PLA/silica mixture with standard deviation (SD).

Silica%	Tensile Strength (MPa)	SD	Toughness (MPa)	SD	Ductility (%)	SD	Elastic Modulus (MPa)	SD	Yield Stress (MPa)	SD
0	62.8	±7.2	3.6	±0.8	11.1	±1.3	839.6	±79.2	52.6	±9.8
5	76.5	±4.1	4.6	±0.5	12.6	±1.7	895.1	±86.4	58.8	±2.0
10	121.0	±13.5	5.6	±0.6	15.3	±1.2	1020.7	±82.1	63.6	±11.6
15	53.9	±8.1	3.1	±0.4	11.4	±1.4	793.2	±94.6	53.0	±8.7
